# Clinical features and management of multifocal hepatic hemangiomas in children: a retrospective study

**DOI:** 10.1038/srep31744

**Published:** 2016-08-17

**Authors:** Yi Ji, Siyuan Chen, Bo Xiang, Zhicheng Xu, Xiaoping Jiang, Xingtao Liu, Qi Wang, Guoyan Lu, Li Yang

**Affiliations:** 1 Division of Oncology, Department of Pediatric Surgery, West China Hospital of Sichuan University, Chengdu, 610041, China; 2 Pediatric Intensive Care Unit, West China Hospital of Sichuan University, Chengdu, 610041, China; 3 Department of Vascular & Interventional Radiology, Chengdu Women and Children’s Central Hospital, Chengdu, 610091, China; 4 Pediatric Intensive Care Unit, West China Second University Hospital, Sichuan University, Chengdu, 610041, China; 5 Department of Radiology, West China Hospital of Sichuan University, Chengdu, 610041, China

## Abstract

Multifocal hepatic hemangioma (MHH) is a benign hepatic tumor that is commonly diagnosed in children with multiple cutaneous infantile hemangiomas (IHs). We present a review of all children with MHH at our institutions. Of the 42 patients, the median age at presentation of MHH was 2.5 months. Thirty-six (85.7%) patients had cutaneous IHs. Twelve (28.6%) patients were symptomatic at presentation. There was no significant association between the number of hepatic hemangiomas and the number of cutaneous IHs. Fourteen (33.3%) patients received some form of treatment for hepatic hemangiomas. The most common type of treatment was oral prednisone in 8 patients, followed by oral propranolol in 6 patients. Two patients were totally resistant to prednisone treatment. They died from congestive heart failure or respiratory distress and coagulopathy. Two patients with problematic facial IH were treated with intralesional triamcinolone injection. The remaining 26 patients were managed with imaging surveillance. On follow-up, all of the survivors had a favorable outcome. Our study suggests that the clinical features of MHH are variable. Our data emphasize the treatment strategy that aggressive treatment is indicated in symptomatic or progressive MHHs, whereas observation management of asymptomatic patients with a few small lesions is safe and appropriate.

Infantile hemangiomas (IHs) are the most common tumors in infants, with an estimated prevalence of 5 to 10%. Populations at increased risk include premature infants, females and Caucasians. IHs are clinically heterogeneous, with an appearance dictated by the location, depth and stage of evolution. Most IHs present as solitary lesions affecting the skin. However, multifocal IHs are not rare and may be associated with extracutaneous involvement, particularly affecting the liver^1^.

Infantile hepatic hemangiomas (HHs) represent the most common hepatic tumors in children. Infantile HHs are vascular neoplasms and should be distinguished from ‘hepatic hemangiomas’ observed in adulthood because the latter are actually venous malformations^1^,^2^. HHs can be classified into three morphologic patterns: focal, multifocal and diffuse. Focal HHs are the hepatic form of cutaneous rapidly involuting congenital hemangiomas and are histologically distinct from IHs^1^. Focal HHs are fully grown at birth and regress faster after birth^3^. In contrast, multifocal and diffuse HHs are true IHs, undergoing parallel phases of growth and involution to cutaneous lesions^2^. Radiographically, multifocal HHs (MHHs) are individual lesions separated by normal intervening liver parenchyma whereas diffuse HHs are characterized by extensive replacement of the hepatic parenchyma with innumerable lesions^1^,^4^,^5^.

Clinically, MHHs are detected more often by abdominal ultrasound (US) in patients associated with multiple cutaneous IHs. Although histologically benign, MHHs have a varied presentation. In many cases, they are clinically silent and self-limiting whereas others can grow dramatically and compress tissue, impair function, and even threaten the patient’s life. Particularly, a subset of patients with MHHs has high-volume arteriovenous or portovenous shunting, resulting in high-output cardiac failure. Furthermore, hypothyroidism can occur in some children with multifocal lesions. Undetected hypothyroidism has been shown to impair the developing central nervous system during the first year of life and can result in permanent neurologic damage. Hypothyroidism can also decrease cardiac output due to impaired contractility^6^. In addition, true HH lesions likely exist in a continuum. Evidence suggests that undetected multifocal lesions can proliferate and evolve into diffuse HH^2^. Children with diffuse lesions are more likely to have a serious clinical course, with a greater risk of morbidity and mortality.

In the present study, we retrospectively evaluated and analyzed all children with a diagnosis of MHHs in 3 hospitals over a period of 15 years. The clinical features of the MHHs are extensively described. The management and outcome of all of the patients are obtained. Furthermore, new concepts emerging in this area of research are also discussed.

## Method

This study was approved by the Institution Review Board of the West China Hospital of Sichuan University, Chengdu Women and Children’s Central Hospital and West China Second University Hospital. We conducted a retrospective review of all of the children with MHH diagnosed from January 2000 to December 2014. The study followed the tenets of the Declaration of Helsinki for research involving human subjects. Written informed consent was obtained from all patients’ parents. All of the children with clinical history and radiographic imaging consistent with MHH were included. MHHs were characterized as multiple tumors with intervening segments of normal hepatic parenchyma. Focal and diffuse HHs were excluded. Clinical records were independently reviewed by two authors. Clinical information, including demographics, clinical presentation, laboratory results, imaging results, treatment, follow-up examinations and outcome, was obtained. In addition to obtaining US results, the available radiology database was queried for computed tomography (CT) scans and magnetic resonance imaging (MRI). Imaging files were reviewed by two senior radiologists with extensive experience in the imaging characteristics of HHs.

We divided patients into 2 groups depending on the number of cutaneous IHs: patients with four or fewer (or without) cutaneous IHs and patients with five or more cutaneous IHs. Statistical analyses of the study were conducted using SPSS 16.0 for Windows (SPSS Inc, Chicago, USA). Student’s *t*-test was used to analyze the quantitative data. The chi-squared test was used for the analysis of categorical data. *P* values less than 0.05 were considered significant.

## Results

Forty-six patients with a diagnosis of MHH were identified. Four patients were excluded because of insufficient clinical information. Of the remaining 42 patients, the median age at presentation of MHH was 2.5 months (range 0.5 to 6.0 months). There were 11 males and 31 females, with a male-to-female ratio of 1:2.8. Twelve (28.6%) patients were premature infants (Table 1).

**Figure 1:**
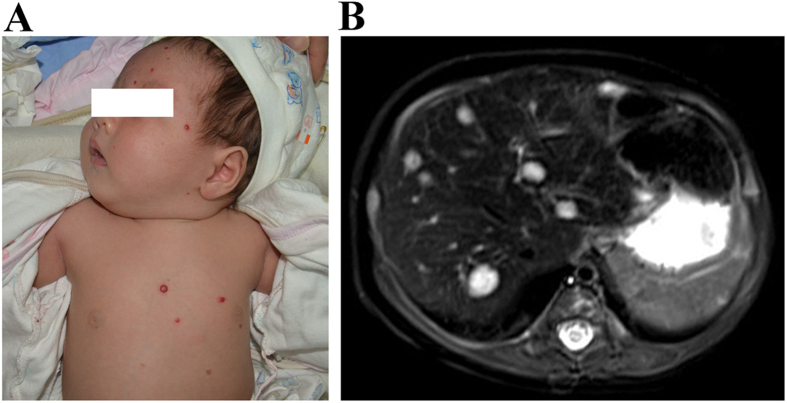
Multiple cutaneous IHs associated with MHHs in a 1-month-old girl. (**A**) Multiple IHs on the face and trunk. This patient also had additional lesions on the extremities. (**B**) T2-weighted axial MRI of the liver showed lesions were hyperintense with intervening areas of normal hepatic parenchyma.

In total, 36 (85.7%) patients had cutaneous IHs. Twenty-seven (64.3%) patients had five or more cutaneous IHs. No differences were noted between patients with four or fewer cutaneous IHs and patients with five or more cutaneous IHs in age at diagnosis and gender distribution (*P* > 0.05). Irrespective of cutaneous IHs, Twelve (28.6%) patients had symptoms. The most common symptom was hepatomegaly, followed by abdominal distention and vomiting. US is the first imaging modality in all cases. Computed tomography (CT) and magnetic resonance imaging (MRI) are frequently used as well (Fig. 1). The hepatic tumor ranged from 0.3 to 4.0 cm in size. Thirteen patients had 10 or more HHs. There was no significant association between the number of HHs and the number of cutaneous IHs (*P* > 0.05). Liver arteriovenous or portovenous shunting was detected in 7 patients. Thirty-seven (88.1%) patients had a baseline echocardiogram performed. Cardiomegaly was present in 6 patients, while 3 patients had heart failure. Anemia was presented in 3 patients. One patient had documented thrombocytopenia. All of the patients had normal liver function test results, except 1 patient with an increase in serum transaminases (the serum aspartate aminotransferase level and serum alanine aminotransferase level were 212 and 179 U/L, respectively). Twenty patients underwent thyroid function studies and two patient had mild hypothyroidism: the serum thyroid-stimulating hormone (TSH) level was 11.7 and 13.2 mIU/L (normal value appropriate for age 0.4–8.2 mIU/L), and serum free thyroxine (fT3 and fT4) levels were normal.

**Figure 2:**
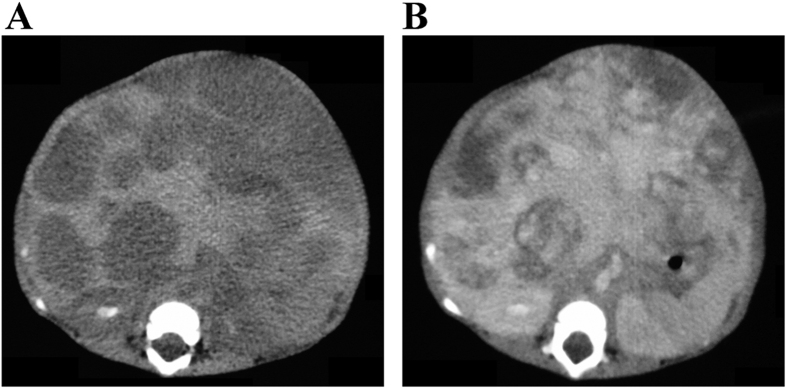
MHH in a 4.5-month-old boy (Patient #2 in Table 2). (**A**) An axial unenhanced CT scan showed multiple hypodense hepatic masses ranging from 1.0 to 4.0 in diameter. (**B**) An axial enhanced CT scan showed an enhancement of the liver masses. Some of the masses showed confluence.

Patients with symptoms, abnormal cardiac function and/or thyroid function, liver arteriovenous or portovenous shunting and dramatic lesion progression received a recommendation to undergo treatment. Fourteen (33.3%) patients received some form of treatment for HHs (Table 2). The most common type of treatment was oral prednisone (2–6 mg/kg), which was given to 8 patients for an average time of 7 months (1.0 to 10 months). Four of these patients responded well to prednisone, and treatment was tapered off within 8 months. Two patients had a partial response to prednisone, and treatment was continued and gradually tapered at 10 and 12 months, respectively. Two patients were totally insensitive to prednisone. Sequential echocardiograms demonstrated persistent ventricular dilatation and worsening cardiac failure. Repeat thyroid function tests showed no evidence of hypothyroidism. In one patient, treatment with vincristine was then started, without any significant improvement. The patient died from congestive heart failure (CHF) (Fig. 2). Because of increased arteriovenous shunting, another patient underwent hepatic embolization at the age of 4.5 months. This procedure significantly improved cardiac function but did not inhibit tumor progression. Finally, the patients died from respiratory distress and coagulopathy.

**Figure 3:**
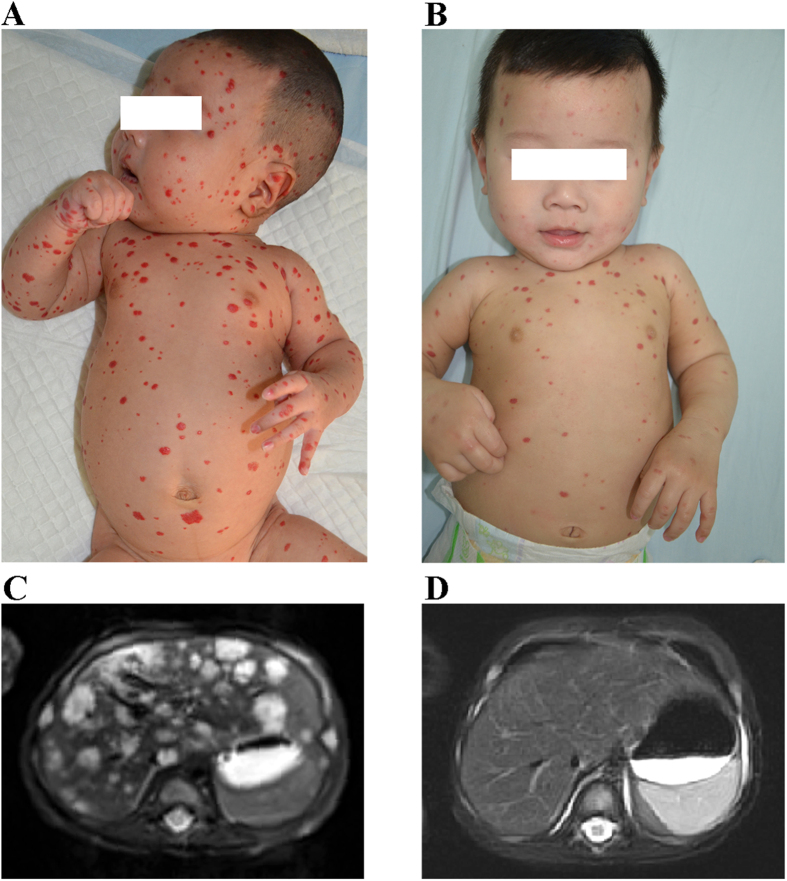
Propranolol treatment for multifocal IH in a 1.5-month-old boy (Patient #11 in Table 2). Clinical photographs of cutaneous IHs, 1 day before propranolol treatment (**A**) and 6 months after the start of treatment (**B**). T2-weighted axial MRI showed MHH, 1 day before propranolol treatment (**C**) and 6 months after the start of treatment (**D**).

Propranolol was administered to 6 patients in a progressive schedule to 2.0 mg/kg per day in 3 daily doses. In two patients with cardiomegaly at the time of diagnosis, their clinical response to propranolol was prompt and documented by the disappearance of echocardiographic signs of cardiac overload within 4 weeks. In another patient with progressive cardiac failure, the use of a combination of spironolactone, digoxin and oral propranolol was required. These treatments significantly improved the cardiac function within 1 week. One patient for whom hypothyroidism was reported clearly demonstrated a tumor type similar to both MHH and diffuse HH (Fig. 3). In this patient, the hepatic hemangiomas were innumerable but they did not entirely replace the hepatic parenchyma. One month after propranolol treatment, the patient became euthyroid and levothyroxine treatment was discontinued. Of the remaining two patients, one had hepatomegaly, and another was asymptomatic. Although they were hemodynamically stable, the dramatic tumor progression was concerning for the eventual onset of CHF. Therefore, propranolol was introduced during the first 3 months of life. After 6 months of treatment, significant regression of the cutaneous hemangiomas and decreases in the number and size of the hepatic lesions were noted in all of the patients.

Two patients with either nodes lesion or lip lesions were treated with a single intralesional triamcinolone injection. They and the remaining 26 asymptomatic patients, all of whom had a few small HHs, were monitored conservatively.

The mean length of follow-up for survivors was 51.1 months (range, 10.0 to 90.0 months). Electrocardiogram, thyroid function tests and abdominal imaging, including US, CT and MRI, were the modalities used to monitor patients. All intrahepatic shunts disappeared with hemangioma regression. Symptom relief was recorded in all of the patients who had undergone treatment. For untreated cases, US was performed monthly until the liver lesion was stable and subsequently every 3 months until there was involution. All of the untreated patients experienced good results on follow-up. Twenty-eight (66.7%) patients had adequate radiographic data to determine time to resolution of hepatic lesions: 6 patients were treated with prednisone (range, 6–90 months), 5 patients were treated with propranolol (range, 5–36 months) and 17 patients were untreated (range, 8–90 months). The time course to compete or nearly complete resolution between prednisone-treated and untreated hepatic lesions was similar between the 2 groups (*P* > 0.05). In contrast, treatment with propranolol resulted in a significantly faster resolution as compared with untreated hepatic lesions (*P* = 0.027).

## Discussion

The pathogenesis of MHH is largely unknown. The risk factors of MHH are also not completely understood. It is recognized that the presence of multiple cutaneous IHs can be associated with MHHs. Among our cases, 64.3% patients had five or more cutaneous IHs. The reason for this association is unknown. An interesting concept is that a greater number of circulating hemangioma stem/progenitor cells may result in the development of IHs in the skin and the liver^7^. Previous studies demonstrated a trend for a greater risk of HHs with increasing numbers of cutaneous IH^8^,^9^. Hughes *et al*.^8^ noted an association between the number of cutaneous IH and the number of HH identified on a screening US. However, our current data argue against this trend. Interestingly, similar results have been obtained by Horii and colleagues, who did not find an association between the number of cutaneous IH and the number of hemangiomas in the liver^10^.

Most MHHs are asymptomatic and are often detected incidentally. MHH patients not detected earlier are most likely identified through abdominal distension or the development of CHF or the progression to diffuse disease, which then presents with hypothyroidism. In this regard, an earlier screening of HHs allows clinicians to follow the appearance of the lesions and institute pharmacologic therapy to prevent tumor progression. However, there is controversy regarding the number of cutaneous IHs that should serve as the threshold at which to perform such screening. Most investigators recommended that infants younger than 6 months of age who present with five or more cutaneous IHs should undergo a screening abdominal US to assess for HHs^10^,^11^. Paradoxically, however, several studies demonstrated that no HHs in patients with between five and nine cutaneous IHs needed treatment^9^,^10^. Vredenborg and colleague suggested that screening should be justified only when 10 or more cutaneous IHs are present^9^. In the present study, more than one-third of our patients are symptomatic and their tumors are detected initially by US. The high rate of symptomatic HH in our cases may reflect a referral bias that many infants enrolled saw pediatric surgeons and probably those with severe HH might have been referred directly to our department. When present, the most common symptom in our cases was hepatomegaly. Remarkably, some of these patients had four or fewer cutaneous IHs. These findings provide evidence that hepatomegaly should be considered as an important bedside clue in diagnosing HH in patients with cutaneous IH. We propose to screen any patient with any number of cutaneous IHs that present with hepatomegaly with an abdominal US.

As mentioned previously, a small number of MHH can lead to death, it is therefore important to identify which factors place patients with MHH at high risk for mortality. However, the accurate identification of appropriate risk factors of critical and fatal outcomes may be difficult because of the small sample size of HH. A few studies have attempted to estimate the risk factors of mortality in patients with HH. Rialon *et al*.^12^ demonstrated that patients with CHF had a higher risk of mortality and shorter survival times. In our study, CHF was present in 3 patients and two of these patients died. In contrast, no patients without CHF died. These observations suggest that CHF can be a predictor of mortality in patients with MHHs. In addition, pathophysiology, such as hypothyroidism, coagulopathy and respiratory distress may potentially coexist in patients with these lesions. Recently, treatment-resistant coagulopathy has been reported as a predictor of fatal outcome, especially if it is symptomatic^13^.

Infantile HH can be associated with consumptive hypothyroidism due to the overproduction of type 3 iodothyronine deiodinase, which deactivates thyroid hormones^14^. Past efforts have revealed that hypothyroidism was more frequently seen in patients with diffuse HHs rather than in those with multifocal lesions^1^,^2^,^6^. In the present study, hypothyroidism was observed in only two patients. Remarkably, hepatic lesions in one patient with hypothyroidism were revealed to have similarities to both multiple and diffuse HHs. These observations further support the concept that there is a missing link between MHH and diffuse HH^2^. In other words, these MHHs could potentially enlarge and coalesce to become diffuse. In such cases, early intervention may be justified to potentially prevent life-threatening progression and reduce associated complications.

To decide whether to treat a patient with MHH, the risks, benefits, and alternatives associated with each of these choices and with each potential intervention are assessed. Most MHHs are uncomplicated and are not likely to become diffuse lesions. The practice of initial observation or ‘wait-and-see’ is reasonable for such lesions. The results from our study support the fact that IHs of the liver – like those of the skin – can be asymptomatic and may not require any therapy, even when multifocal hepatic lesions are present. Alternatively, for HHs that require treatment, the ideal time to begin treatment may be before evidence of complications or sequelae develops. Therapy initiated before or during the early proliferative phase is more likely to be effective in controlling the growth of the lesion or preventing complications^15^. Unfortunately, there is no way to predict the size or severity that proliferative HHs can reach or whether to expect complications. We advocate that patients with symptomatic multifocal hepatic lesions or patients with lesions that dramatically progressed should be aggressively treated. In addition, it is prudent for pediatric providers to reexamine frequently, as often as weekly or monthly, those children with lesions at high risk of causing functional changes. In the current series, the prognosis and outcome of asymptomatic patients with dramatic tumor progression after pharmacologic therapy were promising. These data further support the concept that earlier evaluations and interventions improve outcomes.

For a long time, corticosteroid therapy has been considered the gold standard for controlling the growth and complications of HHs. Although corticosteroid therapy is efficacious, adverse effects, such as Cushingoid facies, personality changes, gastric irritation, osteoporosis and immunosuppression, are frequent^16^. In addition, the possibility of corticosteroid resistance and treatment failure is also remarkable. It has been reported that 23.1% of HH patients were totally insensitive to steroids^13^. Other than corticosteroids, pharmacologic agents such as vincristine, cyclophosphamide and interferon-alpha have also been reported for the treatment of HH^17^. Large intrahepatic shunts in patients presenting with cardiac failure may be considered candidates for embolization^18^,^19^. Of note, the two patients who died in the present series were initially treated with prednisone, but both of them demonstrated an insufficient response. Although they further received vincristine or embolization, these treatment approaches failed to inhibit tumor progression. Therefore, in certain cases, the prompt application of the appropriate therapy is necessary to promote tumor involution and control the life-threatening critical conditions.

First described by Leaute-Labreze *et al*.^20^, propranolol is now the preferred systemic therapy for problematic IHs. Propranolol is effective not only in decreasing and ceasing the growth of IHs but also in prompting a more rapid involution^21^. The therapeutic effect of propranolol is thought to originate from a promotion of pericyte-mediated vasoconstriction^22^. This medication may be useful in clinical practice to hasten IH involution, particularly in cases involving hemodynamic compromise^23^. In recent case studies, rapid responses of severe HHs to propranolol were reported in patients who presented with multifocal or diffuse hepatic lesions and in patients who were initially treated with traditional medical therapy, without significant improvement^24^,^25^,^26^. In the present study, all of the reported patients responded well to propranolol, and all of them experienced rapid radiographic improvement. Remarkably, a dramatic and quick response has especially been noted in patients with hemodynamic compromise or hypothyroidism. Our data suggested that propranolol was effective in both decreasing and ceasing growth of the HH as well as prompting more rapid involution, a combination of findings not typically found in traditional therapies (e.g., corticosteroids). Although prospective studies must be conducted to further evaluate the efficacy and safety of propranolol in the treatment of MHH, current data suggest that propranolol carries promise as a first-line therapy or as part of a multidisciplinary approach.

## Conclusions

The clinical features of MHH are variable and can be non-specific. Screening may allow earlier treatment before life-threatening progression in a subset of children with MHHs, preventing complications and reducing mortality. Active intervention is indicated for symptomatic patients and in cases of dramatic tumor progression. Although prospective data are lacking, our current practice suggests that propranolol should be considered as a first-line therapy or as part of a multidisciplinary approach in MHH patients needing treatment. In addition, our data emphasizes that a therapeutic approach of conservative management of asymptomatic patients with a few small lesions is safe and appropriate.

## Additional Information

**How to cite this article**: Ji, Y. *et al*. Clinical features and management of multifocal hepatic hemangiomas in children: a retrospective study. *Sci. Rep.* **6**, 31744; doi: 10.1038/srep31744 (2016).

## Figures and Tables

**Table 1:** Demographics and clinical characteristics of children with MHHs.

Variables	≤4 Cutaneous IHs	≥5 Cutaneous IHs	Total	*P*-values
	*n* = 15	*n* = 27	*n* = 42	
Age at diagnosis (month)*,†	2.0 (1.5–3.0)	2.1 (1.5–3.0)	2.1 (1.5–3.0)	0.65
Gender‡				1.00
Male	4 (26.7)	7 (25.9)	11 (26.2)	
Female	11 (73.3)	20 (74.1)	31 (73.8)	
Gestational age‡				0.49
Term born	3 (20.0)	9 (33.3)	12 (28.6)	
Born prematurely	12 (80.0)	18 (66.7)	30 (71.4)	
Hepatomegaly‡				0.72
Yes	3 (20.0)	8 (29.6)	11 (26.2)	
No	12 (80.0)	19 (70.4)	31 (73.8)	
Congestive heart failure‡				1.00
Yes	1 (6.7)	2 (7.4)	3 (7.1)	
No	14 (93.3)	25 (92.6)	39 (92.9)	
Hypothyroidism‡				0.86
Yes	0 (0)	2 (7.4)	2 (4.8)	
No	12 (80.0)	19 (70.4)	31 (73.8)	
Unknown	3 (20.0)	6 (22.2)	9 (21.4)	
Number of MHH lesions‡				0.43
2–4	8 (53.3)	9 (33.3)	17 (40.5)	
5–9	4 (26.7)	8 (29.6)	12 (28.6)	
≥10	3 (20.0)	10 (37.0)	13 (30.9)	
Liver arteriovenous shunt‡				0.69
Yes	2 (13.3)	6 (22.2)	8 (19.0)	
No	13 (86.7)	21 (77.8)	34 (81.0)	
Treatment for MHH‡				0.09
Yes	3 (20.0)	11 (40.7)	14 (33.3)	
No	12 (80.0)	16 (59.3)	28 (66.7)	
Mortality‡				0.53
Yes	0 (0)	2 (7.4)	2 (4.8)	
No	15 (100)	25 (92.6)	40 (95.2)	
Duration of follow-up (month)†	44.0 (30.0–70.0)	48.0 (36–72.0)	48.0 (36.0–70.1)	0.50

^*^Age at diagnosis was defined as the date multifocal hepatic hemangiomas were first confirmed by imaging.

^†^Values are presented as a median (interquartile range).

^‡^Values are presented as a number (percentage).

MMH: multifocal hepatic hemangioma; IH: infantile hemangioma.

**Table 2:** Clinical features of 16 treated patients presenting with MHHs.

Cases	No. of skin lesions	Sex	Age at diagnosis	Treatment for IHs	Follow-up/outcome
1	None	Female	3.5 m	Oral prednisone	61 m
2	None	Female	3.0 m	Oral propranolol	40 m
3	1	Female	5 m	Oral propranolol	26 m
4	4	Male	3 m	Intralesional triamcinolone injection	60 m
5	6	Male	4.5 m	Oral prednisone and vincristine	Died from CHF
6	7	Female	1.8 m	Oral prednisone	84 m
7	8	Female	2 m	Intralesional triamcinolone injection	48 m
8	11	Female	2.2 m	Oral propranolol	30 m
9	16	Male	3.0 m	Oral prednisone, embolization	Died from respiratory distress and coagulopathy
10	21	Male	5.0	Oral prednisone	60 m
11	30–100	Female	1.0 m	Oral propranolol	52 m
12	30–100	Male	1.2 m	Oral prednisone	72 m
13	30–100	Female	1.8 m	Oral prednisone	80 m
14	Innumerable	Female	2 m	Oral prednisone	90 m
15	Innumerable	Male	1. 5 m	Oral propranolol	32 m
16	Innumerable	Female	2.1 m	Oral propranolol	38 m

MMH: multifocal hepatic hemangioma; IH: infantile hemangioma; m, month; CHF: congestive heart failure.
